# Knowledge of Avian Influenza (H5N1) among Poultry Workers, Hong Kong, China

**DOI:** 10.3201/eid1712.110321

**Published:** 2011-12

**Authors:** Jean H. Kim, Fung Kuk Lo, Ka Kin Cheuk, Ming Sum Kwong, William B. Goggins, Yan Shan Cai, Shui Shan Lee, Sian Griffiths

**Affiliations:** The Chinese University of Hong Kong, Hong Kong, People’s Republic of China (J.H. Kim, F.K. Lo, M.S. Kwong, W.B. Goggins, Y.S. Cai, S.S. Lee, S. Griffiths);; University of Oxford, Oxford, UK (K.K. Cheuk)

**Keywords:** avian influenza virus (H5N1), influenza, viruses, knowledge, attitudes, practices, preparedness, occupational risk, risk factors, poultry workers, epidemiology, zoonoses, Hong Kong, China

## Abstract

In 2009, a cross-sectional survey of 360 poultry workers in Hong Kong, China, showed that workers had inadequate levels of avian influenza (H5N1) risk knowledge, preventive behavior, and outbreak preparedness. The main barriers to preventive practices were low perceived benefits and interference with work. Poultry workers require occupation-specific health promotion.

In 1997, a zoonosis in humans caused by a highly lethal strain of avian influenza virus (H5N1) was reported in Hong Kong. Live-poultry markets were the source of this outbreak (*1*). As one of the world’s most densely populated regions (16,000 persons/mile^2^ [>6,300 persons/km^2^]) (*2*), Hong Kong is a city at high risk for a large-scale outbreak of avian influenza caused by live poultry in large-volume wholesale markets and within neighborhood wet markets (open food stall markets).

Because members of the average household in Hong Kong shop in wet markets on a habitual basis, these markets are located in the most densely populated areas (Figure) and are commonly multistory complexes or in basement levels of shopping centers. Because poultry workers are a potential bridge population (*3**,**4*), the government has instigated voluntary avian influenza training since 2001 that reviews regulations for workplace disinfection, waste disposal, poultry storage, and personal hygiene measures (*5**,**6*).

**Figure:**
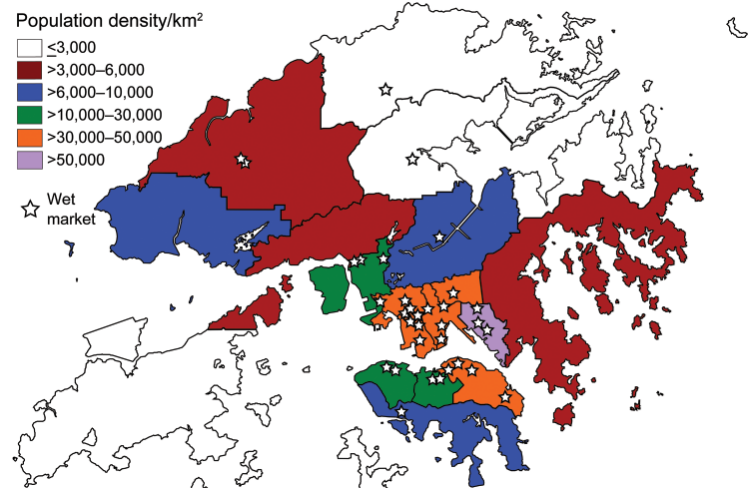
Location of live poultry wet markets (open food stall markets) in relation to population density, Hong Kong, China, June–November 2009.

Despite occupational risk for exposure to avian influenza (*7**,**8*), there have been few studies of poultry workers (*8**–**12*). Most studies were conducted in rural settings in developing countries (*9**–**12*), but findings cannot be readily extrapolated to cities such as Hong Kong because of differences in food-handling practices and occupational settings. Knowledge, perceptions, and work practices of live-poultry workers in Hong Kong have not been examined. Therefore, a survey of these workers is timely and warranted, given confirmed persistence of avian influenza in Asia (*13*).

## The Study

An anonymous, cross-sectional survey was conducted during June–November 2009. Interviewers approached 132 licensed live-poultry retail businesses in wet markets and 23 wholesale establishments. The final sample was 360 poultry workers (194 retailers and 166 wholesalers; response rate 68.1%).

Respondents were asked about their demographics, past month’s work and preventive behavior, and avian influenza–related knowledge on the basis of a World Health Organization factsheet (*14*). We asked perception questions based on the Health Belief Model and the likelihood of adopting certain behavior patterns in the event of a local bird-to-bird or bird-to-human outbreak of avian influenza.

Summative scores were computed for avian influenza–related knowledge, current preventive behavior patterns, outbreak preparedness, and various perception domains. Higher scores reflected more beneficial levels of each domain. Unconditional multilevel regression indicated no evidence of clustering effect by poultry market. Standard multivariable linear regression was conducted by using SAS version 9.1.3 (SAS Institute, Cary, NC, USA) with knowledge, practice, and preparedness scores as outcomes and potential predictors showing p<0.25 in unadjusted analyses as input variables. Distribution of standardized residuals and their association with predicted values were examined to assess model assumptions.

Most (208, 60.1%) respondents were men 35–54 years of age, of whom 192 (55.3%) had worked a mean of 16.1 years in the poultry industry. Respondents showed low mean summative scores for knowledge of avian influenza (Table A1). Nearly two thirds (232, 64.1%) of poultry workers reported that avian influenza virus (H5N1) infects wild birds, but fewer workers reported that this virus could infect live poultry (212, 60.1%), domesticated birds (159, 44.8%), or humans (178, 50.0%).

A total of 242 (69.1%) workers reported that consuming undercooked poultry could transmit the virus, and 210 (59.7%) knew that infection could result from touching bird feces. For other transmission routes, awareness was lower, ranging from 14.0% (48) for eating undercooked eggs to 29.1% (102) for slaughtering poultry.

Ninety-six (27.4%) workers were unsure whether avian influenza virus (H5N1) infection had occurred in humans in Hong Kong, 198 (58%) incorrectly believed that nearly everyone survives this infection, and 110 (32.8%) incorrectly believed that a human vaccine for avian influenza was available. Most (208, 89.9%) respondents were familiar with influenza-like symptoms of avian influenza virus (H5N1) infection such as fever, but fewer workers were aware of respiratory and gastrointestinal symptoms of virus infection.

The Internet and other sources (e.g., health talks) of information about avian influenza were strong independent predictors of greater knowledge. However, training did not result in higher knowledge levels.

Poultry workers reported low-to-moderate levels of compliance with hand hygiene and other preventive measures (ranging from 7.3% [36] using eye protection to 65.2% [245] using handwashing with soap after slaughtering poultry). Working in the poultry industry ≥10 years, lower perceived barriers to preventive behavior, and retail poultry work were independent predictors of higher preventive behavior scores.

With regard to avian influenza–related perceptions, lack of training (277, 83.4%) and the view that compliance with all infection regulations is difficult during peak hours (218, 64.9%) were the most frequently cited barriers to adoption of preventive behavior. A total of 154 (46.4%) workers believed that face masks reduced business, and 153 (46.1%) believed that vaccination was expensive.

Low anxiety about illness was reported by 242 (76.6%) respondents. In the event of a local outbreak, workers expressed various levels of acceptance for precautionary actions, ranging from 15.8% (56) for reducing work hours to 82.4% (290) for seeking medical care for influenza-like symptoms. Ninety-six (27.4%) respondents anticipated taking oseltamivir. Greater perceived benefit of preventive behavior was the strongest independent predictor of higher preparedness scores (Table A2).

## Conclusions

Similar to other regions (*8**–**11*), poultry workers in Hong Kong showed low risk perceptions for avian influenza, inadequate knowledge, and a wide range of compliance with preventive measures. Because training (*6*) was not associated with overall preventive behavior or preparedness, there may be an unmet need for occupation-specific health information.

Higher levels of knowledge demonstrated by workers who accessed health information sources (e.g., Internet) that provide detailed information suggest that comprehensive, occupation-relevant information should be more widely accessible. However, occupational practices of animal workers might not be amenable to change solely on the basis of improvements in knowledge. Only 129 (42.1%) respondents reported that poultry workers could realistically adhere to all government guidelines (*6*). Interference with work, high cost, and reduction of business were repeatedly cited as impediments to the adoption of preventive behavior. Even in the event of local outbreaks of avian influenza, most workers were not amenable to actions having adverse economic effects such as reducing work hours. Animal workers are thereby unlikely to widely adopt preventive behavior if these measures conflict with their economic interests.

Despite the ongoing government regulations regarding avian influenza in Hong Kong (*6*), a complete ban on live poultry is unrealistic because of the culturally entrenched demand for fresh poultry. Increasing knowledge and risk perceptions while simultaneously reducing occupational barriers to preventive behavior thereby continues to be the cornerstone of effective zoonotic infection control among animal workers.

Implications of these findings extend to other poultry-borne pathogens, such as *Campylobacter* spp. and *Salmonella* spp., which share common preventive measures. Close adherence to workplace measures will likely reduce outbreak risk for other poultry-borne diseases. Therefore, a framework for greater integration of risk management strategies and worker education of these poultry-borne infections tailored to the local context is worthwhile and cost-effective.

In the spirit of the One Health Commission, which calls for an integrated, interdisciplinary approach to human–veterinary–environmental health challenges (*15*), the fight against global pandemics, such as those of avian influenza virus (H5N1), necessitates greater dialogue and collaborative leadership between governments and livestock industries. Development of realistic occupational safety measures is an ongoing challenge for national governments.

## Appendix

**Table A1:** Responses to questionnaire Items related to knowledge and preventive practices for avian influenza (H5N1) for 360 poultry workers, Hong Kong, China*

Item†	Value
Virus can infect	
Wild birds? [Y] / Live poultry [Y] / Domestic birds? [Y]	65.4/60.1/44.8
Humans? [Y] / Other animals? [Y]	50.0/17.0
Virus can be transmitted from 1 place to another on	
Bird feces? [Y] / Poultry cages? [Y] / Bird feed? [Y] / Clothing and shoes? [Y]	82.7/26.8/16.9/11.9
Humans can be infected by	
Eating behavior: eating poultry cooked well done? [N] / Eating runny eggs? [Y]	69.1/14.0
Infected bird contact: bird feces? [Y] / Breathing near birds? [Y] / Swimming with birds? [Y]	59.7/22.1/19.4
Occupational risks: slaughtering poultry? [Y] / Defeathering poultry? [Y]	29.1/14.9
Symptoms of infection in humans	
Dermatologic: Hair loss? [N] / Rash? [N]	99.4/98.2
Influenza-like: Fever? [Y] / Cough? [Y] / Muscle pain? [Y] / Runny nose? [Y]	89.8/67.1/67.1/54.5
Respiratory: Difficulty in breathing? [Y] / Crackling breathing sounds? [Y]	59.2/18.8
Other: Vomiting? [Y] / Diarrhea? [Y] / Nose bleeding? [Y]	25.0/14.3/2.9
There have been reported human cases	
Somewhere? [Y] / Asia? [Y] / People’s Republic of China? [Y] / Hong Kong? [Y]	96.8/91.1/78.2/72.6
100% effective, commercially available vaccines exists	
For birds? [N] / For humans? [N]	80.2/67.2
Duration virus can survive outside body	
<6 h / 6h– 24h / several days / [>1 wk]	29.0/22.7/18.0/30.3
Mortality rate for humans	
Almost everyone survives / <50% / [50%–89% die] / >90% / don’t know	57.9/26.3/13.5/1.2/1.2
Overall knowledge score, range 0–36, mean, SD	6.7, 6.43
Knowledge score multivariable linear regression model, β (95% CI), p value‡§	
Educational level, primary or less = referent, F1–3, >F4	0.97 (0.07–1.87), 0.035
Household monthly income >20,000 Hong Kong dollars	1.61 (0.09–3.13), 0.038
Received prevention information from the Internet	4.35 (2.58–6.13), <0.0001
Received prevention information from other sources¶	3.86 (1.10–6.62), 0.006
In the past month, how frequently did you? Almost always / Sometimes? / Never?	
Handle live chickens with bare hands	37.5/27.5/35.0
Handle dead chickens with bare hands	10.3/27.4/62.3
Wear eye protection when handling chickens	7.3/22.3/70.4
Wear face mask when handling chickens	25.3/35.2/39.5
Wear PPE (e.g., apron, mask) when handling chickens	51.2/22.4/26.4
Sterilize your clothes	52.9/31.8/15.3
Wash hands with soap after killing chickens	65.2/24.6/10.2
Overall preventive practice score, mean ± SD (range)#	8.16 ± 3.26 (0–14)
Practice score multivariable linear regression model, β (95% CI), p value‡**	
>10 y working in the poultry industry; <10 y is referent	1.45 (0.39–2.51), 0.010
Retail shop worker; wholesale is referent	1.11(0.08–2.14), 0.034
Below median perceived barriers score; above median is referent	1.44 (0.43–2.46), 0.006

*Values are % responding correctly unless otherwise indicated. Y, yes; N, no; PPE, personal protection equipment; CI, confidence interval. †Correct answers are indicated in [brackets]. ‡Variance inflation factors (VIF) diagnostics indicated no evidence of colinearity (all VIF <1.2) among variables in final models. Model fit analysis showed that standardized residuals of models were normally distributed and not associated with standardized predicted values. §Final model constant for knowledge score, α (95% CI) 13.70 (11.8–15.6). The following candidate covariates had the following β coefficients and p values before removal from knowledge score model: age, β = 0.25, p = 0.57; <10 years in poultry industry, β = 1.08, p = 0.20; newspaper information source, β = 0.79, p = 0.284; health workers information source, β = −1.06, p = 0.441; poster information source, β = −0.04, p = 0.650. ¶Other health information sources included health talks, seminars, school, radio, flyers, and other poultry workers. #Scored 2 = always, 1 = sometimes, 0 = never for computing summative score. Items 1 and 2 about chickens are reverse coded. **Final model constant for practice score, α (95% CI) 6.18 (5.08–7.29). The following candidate covariates had the following β coefficients and p values before removal from practice score model: monthly income >20,000 Hong Kong dollars, β = −0.036, p = 0.956; above median avian influenza (H5N1) susceptibility score, β = 0.171, p = 0.797; above median avian influenza (H5N1) perceived severity score, β = −0.965, p = 0.143.

**Table A2:** Perceptions of and outbreak preparedness for avian influenza (H5N1) for 360 poultry workers, Hong Kong, China*

Item	Value
Perceived benefits of preventive measures	
Influenza vaccination for poultry	69.8
Handwashing with soap	68.4
Used gloves	59.4
Killed all live poultry in market by end of every day	52.4
Used N95 face masks	38.4
Two wet market rest days a month for cleaning	38.0
Made sure poultry are healthy before buying	31.1
Sterilized cutting boards and surfaces	27.2
Stayed >1 m from live or dead birds	19.2
Took antiviral drugs	14.4
Used goggles	10.1
Perceived benefit summative score, mean ± SD (range)	4.05 ± 2.33 (0–11)
Perceived severity	
Anxiety toward severity of symptoms: low/medium/high	76.6/15.5/7.9
Anxiety toward severity of infection: less than SARS/similar to SARS/more than SARS	46.0/45.4/8.6
Perceived severity summative score, mean ± SD (range)	2.37 ± 1.42 (0–4)
Perceived susceptibility	
Government has sufficient measures to prevent infection in humans	65.8
I have immunity to avian influenza	48.4
Virus is transmitted from birds to humans	32.7
General public is susceptible to avian influenza	15.8
An epidemic will occur in Hong Kong	14.7
Poultry workers are highly susceptible to avian influenza	13.9
Perceived susceptibility summative score, mean ± SD (range)	1.91 ± 1.19 (0–6)
Perceived self-efficacy	
I know how to protect myself from avian influenza	82.4
I can reduce the risk for transmission in the community	76.6
I am confident that I know how to handle infected poultry	48.3
Perceived self-efficacy summative score, mean ± SD (range)	2.05 ± 0.93 (0–3)
Perceived cues to action	
Received prevention information from mass media	93.3
Public announcements are effective reminders of risk behavior	61.2
Exposed to worksite cues of action (health workers, posters, employer)	41.7
Cues to action summative score, mean ± SD (range)	2.04 ± 0.75 (0–3)
Perceived barriers toward preventive measures	
Never received any infection control training	83.4
Following hygiene guidelines is difficult during peak hours	64.9
It is difficult to attend training on prevention	57.6
Wearing face masks when working will reduce business	46.4
Influenza vaccination is too costly	46.1
Wet market does not provide sufficient cleaning facilities	35.3
Influenza vaccination is inconvenient	33.3
Perceived barrier summative score, mean ± SD (range)	3.69 ± 1.66 (0–7)
Preparedness	
Know who to contact for a suspected outbreak at work?	71.1
In the past year, have you been vaccinated for influenza?	28.8
In the event of a local outbreak in birds, are you likely to	
Increase sanitation measures at work	79.7
Wash hands more often	72.6
Accept influenza vaccination	67.5
Prevent customers from direct contact with birds	62.4
Get influenza vaccination	62.2
Wear a face mask during work	57.3
Wear more PPE during work	30.8
Stay away from chickens	24.3
Reduce work until condition improves	15.8
In the event of a small local human outbreak, will you	
See a doctor right away if you have symptoms	82.4
Wash hands more often	68.5
Get influenza vaccination	62.2
Wear a face mask during work	59.4
Wear a face mask in public	38.9
Take oseltamivir	27.4
Stay away from chickens	24.1
Quarantine yourself if you feel sick	17.9
Preparedness summative score, mean ± SD (range)	9.22 ± 3.77 (0–18)
Preparedness score multivariable linear regression model, β (95% CI), p value†	
Above median perceived barriers score; above or equal to median is referent	1.56 (0.64–2.47), 0.001
Above or equal to median perceived susceptibility score; below median is referent	0.98 (0.21–1.75), 0.013
Above or equal to median perceived benefit score; below median is referent	3.42 (2.61–4.22), <0.001
Above or equal to median knowledge score; below median is referent	1.26 (0.46–2.07), 0.002

*Values are % agree/yes unless otherwise indicated. Wet market, open food stall market; SARS, severe acute respiratory syndrome; PPE, personal protection equipment; CI, confidence interval. †Variance inflation factors (VIF) diagnostics indicated no evidence of colinearity (VIF<1.2) among variables in final models. Model fit analysis showed that standardized residuals of models were normally distributed and not associated with standardized predicted values. Final model constant for preparedness score α (95% CI) 5.64 (4.49–6.80); not significant at p<0.05. The following candidate covariates had the following β coefficients and p values before removal from the final backward elimination model: cues to action above median, β = 0.101, p = 0.840; avian influenza (H5N1) training, β = 0.432, p = 0.502; >10 years in poultry industry, β = 0.543, p = 0.253; educational level, β = −0.232, p = 0.390; monthly income >20,000 Hong Kong dollars, β = 0.576, p = 0.226.

## References

[1] World Health Organization. Confirmed human cases of avian influenza A (H5N1), September 6, 2007 [cited 2007 Oct 10]. http://www.who.int/csr/disease/avian_influenza/country/en/index.html

[2] Census and Statistics Department, The Government of the Hong Kong Special Administrative Region. Key statistics of the 2006 population census. Hong Kong: The Government; 2006.

[3] Webster RG. Wet markets: a continuing source of severe acute respiratory syndrome and influenza? Lancet. 2004;363:234–6. 10.1016/S0140-6736(03)15329-9 14738798 PMC7112390

[4] Bridges CB, Lim L, Hu-Primmer J, Sims L, Fukuda K, Mak KH, Risk of influenza A (H5N1) infection among poultry workers, Hong Kong, 1997–1998. J Infect Dis. 2002;185:1005–10. 10.1086/340044 11930308

[5] Hong Kong Food and Environmental Hygiene Department. Prevention of Avian influenza, February 3, 2004 [cited 2011 Jun 1]. http://www.fehd.gov.hk/english/safefood/avian_flu/guide.html

[6] Hong Kong Food and Environmental Hygiene Department. Training for prevention of avian influenza.[in Chinese], June 11, 2006 [cited 2011 Jun 1]. http://www.info.gov.hk/gia/general/200106/11/0611240.htm

[7] Kung NY, Morris RS, Perkins NR, Sims LD, Ellis TM, Bissett L, Risk for infection with highly pathogenic influenza A virus (H5N1) in chickens, Hong Kong, 2002. Emerg Infect Dis. 2007;13:412–8. 10.3201/eid1303.060365 17552094 PMC2725907

[8] Mounts AW, Kwong H, Izurieta HS, Ho Y, Au T, Lee M, Case‐control study of risk factors for avian influenza A (H5N1) disease, Hong Kong, 1997. J Infect Dis. 1999;180:505–8. 10.1086/314903 10395870

[9] Abbate R, Di Giuseppe G, Marinelli P, Angelillo IF. Knowledge, attitudes, and practices of avian influenza, poultry workers, Italy. Emerg Infect Dis. 2006;12:1762–5. 10.3201/eid1211.060671 17283632 PMC3372355

[10] Olsen S, Laosiritaworn Y, Pattanasin S, Prapasiri P, Dowel S. Poultry-handling practices during avian influenza outbreak, Thailand. Emerg Infect Dis. 2005;11:1601–3. 16318704 10.3201/eid1110.041267 PMC3366731

[11] Leslie T, Billaud J, Mofleh J, Mustafa L, Yingst S. Knowledge, attitudes, and practices regarding avian influenza (H5N1), Afghanistan. Emerg Infect Dis. 2008;14:1459–61. 10.3201/eid1409.071382 18760020 PMC2603107

[12] Fasina FO, Bisschop SPR, Ibironke AA, Meseko CA. Avian influenza risk perception among poultry workers, Nigeria. Emerg Infect Dis. 2009;15:616–7. 10.3201/eid1504.070159 19331751 PMC2671418

[13] Indriani R, Samaan G, Gultom A, Loth L, Indryani S, Adjid R, Environmental sampling for avian influenza virus A (H5N1) in live-bird markets, Indonesia. Emerg Infect Dis. 2010;16:1889–95. 21122218 10.3201/eid1612.100402 PMC3294595

[14] World Health Organization. Avian influenza (“bird flu”): fact sheet, February 2006 [cited 2009 Aug 10]. http://www.who.int/mediacentre/factsheets/avian_influenza/en/index.html#birds

[15] One Health Commission. 2009 [cited 2011 Mar 29]. http://www.onehealthcommission.org/summit.html

